# An empirical study of augmented analytics adoption and its impact on firms’ agility through AI-based models

**DOI:** 10.3389/frai.2026.1940124

**Published:** 2026-09-24

**Authors:** Moath Srahin, Ala Mughaid, Mahmoud AlJamal, Abedalmuhdi Almomany, Muhammed Sutcu

**Affiliations:** 1 Department of Information Systems, Yarmouk University, Irbid, Jordan; 2 Department of Computer Science, Gulf University for Science and Technology (GUST), Mishref, Kuwait; 3 Department of Information Technology, Prince Al-Hussein Bin Abdullah II Faculty of Information Technology, The Hashemite University, Zarqa, Jordan; 4 Department of Cybersecurity, Irbid National University, Irbid, Jordan; 5 Electrical & Computer Engineering Department, College of Engineering & Architecture, Gulf University for Science & Technology, Mishref, Kuwait

**Keywords:** artificial intelligence (AI), augmented analytics, competitive advantage (CA), data analysis, firm agility (FA), natural language processing (NLP), PLS-SEM, task-technology fit (TTF)

## Abstract

Augmented analytics extends business analytics by combining artificial intelligence, machine learning, and natural language processing. However, organizations differ in their ability to adopt these technologies and derive value from them. This study examines the organizational conditions associated with augmented analytics adoption and the role of firm agility in linking adoption to competitive advantage. The proposed model draws on Task-Technology Fit, the Resource-Based View, and Dynamic Capability Theory. It includes six resource-technology fit dimensions as antecedents of augmented analytics adoption and tests firm agility as a mediator. Survey data from a final analytic sample of 287 respondents across several business sectors in Jordan were analyzed using Partial Least Squares Structural Equation Modeling (PLS-SEM). The findings indicate that the fit dimensions do not contribute equally to adoption, with data, individual, cultural, and analytics-capability fit showing the clearest relationships. The results also show that the relationship between augmented analytics adoption and competitive advantage operates mainly through firm agility. The study therefore suggests that the value of augmented analytics depends not only on adopting the technology but also on aligning organizational resources and using analytical insights to support timely organizational responses.

## 1 Introduction

Organizations operate in an environment where large volumes of data increasingly shape competition and decision-making. Augmented analytics combines artificial intelligence (AI), machine learning (ML), and natural language processing (NLP) with conventional analytics to automate parts of the analytical process and support the interpretation of complex data. These capabilities can help decision-makers identify patterns, generate predictions, and use data more efficiently. As a result, augmented analytics has become relevant not only to data analysis but also to the way organizations make and implement decisions.

The potential value of augmented analytics goes beyond operational efficiency. Organizations increasingly use data and analytics to support innovation, responsiveness, and competitive positioning. Industry 4.0 and related technologies, including social platforms and the Internet of Things, have increased both the volume and variety of data available to firms (Okwu et al., 2022). When these data are analyzed effectively, they can support strategic decisions and innovation (Ausat et al., 2023; Choi et al., 2022). However, analytics initiatives also face practical problems, including data preparation, interpretation bias, and shortages of employees with the required analytical skills (Sallam et al., 2017; Prat, 2019). These issues make successful adoption an organizational as well as a technological challenge.

Recent developments in AI and machine learning have expanded the range of tasks analytics can support (Lew et al., 2023). Augmented analytics applies these technologies to activities such as data preparation, insight generation, visualization, and interpretation (Richardson et al., 2020). This can help organizations examine past patterns and respond more quickly to new information. At the same time, moving from conventional analytics to AI-supported analytics is not automatic. Implementation depends on the organization’s technological infrastructure, data environment, skills, and other internal conditions (Rahm and Rahm-Skågeby, 2023; Sun and Huo, 2021).

Investment in analytical tools alone is unlikely to be sufficient. Firms also need reliable, integrated data; appropriate technical and analytical skills; and an organizational culture that supports evidence-based decision-making (Bozkus, 2023; Horani et al., 2023; Skourletopoulos et al., 2018; Allioui and Mourdi, 2023). The present study examines these conditions through Task-Technology Fit (TTF), the Resource-Based View (RBV), and Dynamic Capability Theory. TTF explains alignment between technological functions and organizational requirements; RBV explains why complementary data, infrastructure, skills, and routines condition adoption; and Dynamic Capability Theory explains how organizations convert analytical insight into timely sensing, decision-making, and reconfiguration. Together, these perspectives connect technology-resource alignment with both adoption and post-adoption value realization.

This study contributes to this research stream in two ways. It first broadens the scope beyond a single task-technology correspondence by incorporating six separate resource-technology fit dimensions that can enable or restrict adoption. It then extends the analysis beyond the adoption decision itself: complementary resources are expected to support AAA, yet Dynamic Capability Theory suggests that competitive value materializes only when firms translate analytical insight into greater agility. Together, these steps form a theoretical chain—resource-technology alignment leading to augmented analytics adoption, then to firm agility, and ultimately to competitive advantage—whose implications for the empirical findings are taken up in the Discussion.

## 2 Literature review and theoretical background

As organizations grapple with data that keeps growing in volume, variety, and velocity, augmented analytics has steadily drawn more attention. Born out of Fourth Industrial Revolution developments, it folds artificial intelligence (AI), machine learning (ML), and natural language processing (NLP) into established analytical practice to surface patterns, generate insights, and support decision-making (Prat, 2019; Abas et al., 2020; Rouhani et al., 2016; Alghamdi and Al-Baity, 2022; Oesterreich et al., 2021). Understanding what this means organizationally takes more than cataloging technical functions—it also means setting augmented analytics apart from conventional analytics and tracing how its outputs turn into usable business value.

Augmented analytics is best understood as an AI-enabled extension of business intelligence and conventional analytics, not a replacement for them. Where traditional business intelligence typically runs on manually prepared data, predefined queries, dashboards, and analyst-led interpretation, augmented analytics weaves machine learning and natural language technologies through the analytical workflow itself. Its functions span automated data preparation, detection of relationships and anomalies, generation of explanatory or predictive insights, visualization, and natural-language interaction with outputs (Sallam et al., 2017; Prat, 2019; Richardson et al., 2020; LaPlante, 2019; Bharadiya, 2023; Alghamdi and Al-Baity, 2022; Oesterreich et al., 2021). In short, it is defined by the augmentation and partial automation of the analytics lifecycle, not by any particular algorithm or software product.

Earlier work suggests AI-supported analytics can curb certain forms of judgment bias simply by putting more data to use in decision processes (Ghasemaghaei and Calic, 2019). It can also automate parts of data preparation and analysis (Jarrahi, 2018)—functions that may sharpen the speed and consistency of analytical work while reducing some sources of human error (Rouhani et al., 2016).

This distinction also sharpens the role of human decision-makers. Augmented analytics does not eliminate managerial judgment; it reshapes how that judgment gets informed and exercised. Algorithms can comb through large, heterogeneous datasets quickly, surface patterns that would be hard to spot manually, and present findings to users at different levels of analytical expertise. Interpreting organizational context, questioning model outputs, weighing competing objectives, and deciding whether and how to act on an insight, though, remain the responsibility of managers and domain specialists. Augmented analytics is thus best read as a human-AI decision-support arrangement, pairing computational scale with contextual and managerial knowledge (Prat, 2019; Rouhani et al., 2016; Ghasemaghaei and Calic, 2019; Jarrahi, 2018; Alghamdi and Al-Baity, 2022; Salazar and Kunc, 2025).

The literature also points to an expanding role for augmented analytics across business functions. As AI and machine-learning capabilities mature, analytics increasingly supports activities from marketing and customer analysis through to operations and supply-chain decisions. Realizing these benefits, however, hinges on an organization’s ability to integrate new analytical tools with existing systems, maintain data quality, and build the human capabilities needed to interpret and act on what the analysis produces (Davenport, 2018; Eskandarivatannezhad, 2023; Bharadiya, 2022; Alghamdi and Al-Baity, 2022; Oesterreich et al., 2021).

Recent application-oriented research also emphasizes a complementary computational perspective concerning AI-enabled analytics. Reinforcement-learning-based traffic optimization has demonstrated that continuously analyzed operational data can facilitate adaptive, real-time decision-making in complex environments (Almomany et al., 2025). At the implementation stage, FPGA-based acceleration of clustering and machine-learning workloads has illustrated that parallel and reconfigurable architectures can significantly reduce processing times and enhance throughput for computationally demanding analytics (Almomany et al., 2022; Almomani et al., 2026). These findings are pertinent to augmented analytics, as they show that the efficacy of AI-supported insight generation depends not only on the model’s capability but also on whether the underlying computing infrastructure can process data and deliver results at the speed needed for operational decision-making. This technological perspective complements the current study’s focus on IT infrastructure alignment, analytics capability, and organizational agility.

At the organizational level, adoption involves more than installing an analytics platform. Meaningful adoption requires integrating the technology with data sources, work routines, reporting practices, and decision processes. It also requires users to possess the skills and authority needed to interpret and apply analytical outputs. These requirements explain why the present study treats adoption as dependent on several complementary forms of resource-technology fit: infrastructure enables reliable operation; suitable data support credible analysis; individual and analytical capabilities permit effective use; task alignment connects functionality to work requirements; and cultural alignment supports evidence-based action (Eskandarivatannezhad, 2023; Arunachalam et al., 2018; Côrte-Real et al., 2017; Muslimin et al., 2017; Ashrafi et al., 2019; Ammenwerth et al., 2006; Ghasemaghaei et al., 2017; Daradkeh, 2019; Lee et al., 2007; Warth et al., 2011; Alghamdi and Al-Baity, 2022; Oesterreich et al., 2021).

Analytical capabilities have repeatedly been tied to organizational competitiveness: firms use them to track market shifts, read customer preferences, and inform strategic decisions (Zvarikova et al., 2022; Davenport, 2018). How effective they are, though, hinges on data quality, systems integration, employee skill, technology investment, and organizational support for data-driven decision-making (Eskandarivatannezhad, 2023).

As AI’s footprint in analytics grows, the key question is no longer just what the technology can do—it is what conditions support its adoption, and through what organizational mechanisms that adoption connects to business outcomes (Bharadiya, 2022).

Ultimately, the business value of augmented analytics hinges on a conversion process. Faster or automated insight generation carries limited strategic value unless organizational processes can actually respond to what the analysis reveals. Firms that embed analytical insight into coordinated, timely decision-making can catch changes earlier, weigh alternatives faster, and reconfigure activities as opportunities or threats emerge—the conceptual bridge running from augmented analytics adoption to firm agility, and through agility, to competitive advantage (Davenport, 2018; Ashrafi et al., 2019; Ghasemaghaei et al., 2017; Seddon et al., 2017; Wamba et al., 2015; Salazar and Kunc, 2025; Hyun et al., 2023). This is also why the study examines both what precedes adoption and the post-adoption mechanism through which analytical capability becomes organizational value.

Recent literature further supports this line of reasoning. A growing body of work on AI, business analytics, and digital transformation now treats organizational agility as a key capability for coping with technological and market change (Atienza-Barba et al., 2024; Xu et al., 2024; Abdul Wahab and Radmehr, 2024; Mariani and Mancini, 2025; Sadeghi R et al., 2024; Salazar and Kunc, 2025; Hyun et al., 2023), pointing to data-related capabilities, human-AI complementarity, and agility as the mechanisms that turn digital and AI investment into organizational outcomes.

The study accordingly retains its original focus on the antecedents of augmented analytics adoption while paying closer attention to what follows it. Resource-technology fit is treated as a condition for adoption, and firm agility as the mechanism that links adoption to competitive advantage.

### 2.1 Task-technology fit and resource alignment

Task-Technology Fit (TTF) captures how well a technology supports the tasks users need to carry out. Goodhue and Thompson (1995) define tasks as the activities through which individuals turn inputs into outputs, with fit denoting the degree of match between task requirements and technology characteristics. Within information-systems research, TTF thus centers on whether a system supplies the functions and information required for effective task performance (Goodhue and Thompson, 1995; Goodhue, 1998).

Conventional TTF research asks, at its core, whether a technology’s functions and information outputs match the tasks users must perform (Goodhue and Thompson, 1995; Goodhue, 1998). That question still matters for augmented analytics, where relevant capabilities—automated insight generation, self-service visualization, integration with data sources—must align with task demands such as decision speed, data heterogeneity, and the level of analytical detail required. The present model builds on this individual task-function logic but does not stop there: adoption may depend on six separate forms of resource-technology alignment rather than on task fit alone.

The Resource-Based View contributes an organizational-level explanation. Firms differ, it holds, because they possess and deploy heterogeneous resources—particularly resource combinations that are both valuable and hard to imitate (Barney, 1991). In the present context, infrastructure, data, user expertise, culture, and established analytical routines act as complementary resources that make the same augmented-analytics technology easier or harder to adopt. From this view, simply acquiring an analytical platform does not, by itself, guarantee assimilation or value.

Dynamic Capability Theory pushes the argument a step further, beyond the adoption decision itself. Dynamic capabilities are what allow organizations to sense change, seize opportunities, and reconfigure their activities as conditions shift (Teece, 2007). In the proposed model, AAA broadens the speed and reach of analytical sensing, while firm agility turns those insights into timely decisions and action. Taken together, the three perspectives add explanatory power across a sequence that none covers alone: resource-technology alignment supports adoption, and agility links that adoption to competitive advantage (Figure 1).

**Figure 1:**
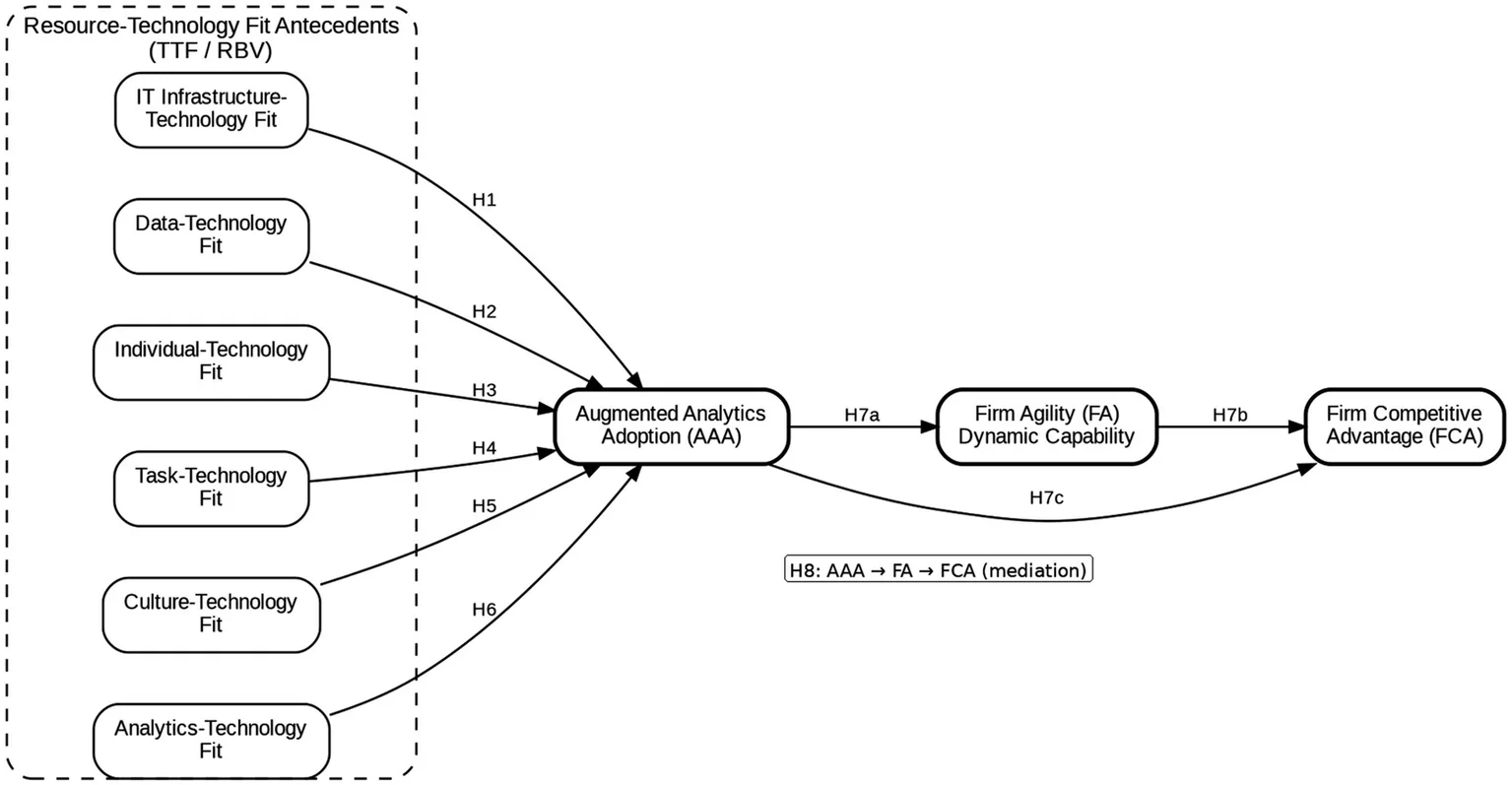
Research model and hypothesized relationships, including the H8 mediation pathway.

### 2.2 Research model and hypotheses

*H1*: IT infrastructure-technology fit has a positive effect on augmented analytics adoption.

*H2*: Data-technology fit has a positive effect on augmented analytics adoption.

*H3*: Individual-technology fit has a positive effect on augmented analytics adoption.

*H4*: Task-technology fit has a positive effect on augmented analytics adoption.

*H5*: Culture-technology fit has a positive effect on augmented analytics adoption.

*H6*: Analytics-technology fit has a positive effect on augmented analytics adoption.

*H7a*: Augmented analytics adoption has a positive effect on firm agility.

*H7b*: Firm agility has a positive effect on firm competitive advantage.

*H7c*: Augmented analytics adoption has a positive direct effect on firm competitive advantage.

*H8*: Firm agility mediates the relationship between augmented analytics adoption and firm competitive advantage.

## 3 Methodology

### 3.1 Instrument development

This study uses a quantitative, theory-driven design. Six fit dimensions—IT infrastructure-technology fit, data-technology fit, individual-technology fit, task-technology fit, culture-technology fit, and analytics-technology fit—are modeled as antecedents of Augmented Analytics Adoption (AAA). The model then examines the relationships between AAA and firm agility (FA) and firm competitive advantage (FCA), including FA’s mediating role. The hypotheses were tested using survey data and Partial Least Squares Structural Equation Modeling (PLS-SEM) in SmartPLS.

The empirical analysis follows two linked stages. First, the measurement model is examined to assess the quality of the reflective and formative measurement specifications. Second, the structural model is assessed to test the hypothesized paths, explained variance, effect sizes, and the mediating role of firm agility. The measurement model, structural model, mediation, *R^2^*, and *f^2^* results are drawn from one traceable 287-case SmartPLS analysis. The blindfolding-based *Q^2^* assessment was subsequently recomputed from the same archived 287-case standardized indicator matrix using the same PLS model specification, as described in Section 3.6.

Reflective and formative measurement must be distinguished. Reflective indicators are treated as manifestations of an underlying construct and are evaluated primarily through indicator reliability, internal consistency, convergent validity, and discriminant validity. Formative indicators, in contrast, contribute to the formation of the construct and are evaluated through collinearity, outer weights, significance, and substantive relevance (Leguina, 2015). In this study, the fit constructs, firm agility, and competitive advantage are specified reflectively, whereas Augmented Analytics Adoption (AAA) is specified formatively (Table 1).

**Table 1:** Summary of constructs, definitions, and references.

Construct	Definition	References
IT Infrastructure-Technology Fit (IT-INF)	Degree of alignment between augmented analytics technology and the quality/readiness of the firm’s IT infrastructure.	Arunachalam et al. (2018); Côrte-Real et al. (2017); Muslimin et al. (2017)
Data-Technology Fit (DTF)	Extent to which data of appropriate quality, accessibility, and suitability for reporting and analysis tasks match augmented analytics technology.	Skourletopoulos et al. (2018); Ghasemaghaei and Calic (2019); Ashrafi et al. (2019)
Individual-Technology Fit (ITF)	Degree of alignment between augmented analytics technology and individual analytical and technical capabilities.	Muslimin et al. (2017); Ammenwerth et al. (2006); Ghasemaghaei et al. (2017)
Task-Technology Fit (TTF)	Extent to which augmented analytics technology meets business users’ reporting and analytics task requirements.	Daradkeh (2019); Goodhue and Thompson (1995); Goodhue (1998)
Culture-Technology Fit (CTF)	Degree of correspondence between augmented analytics features and the firm’s cultural characteristics.	Lee et al. (2007)
Analytics-Technology Fit (ATF)	Extent to which augmented analytics technology meets the organization’s analytical capability requirements.	Ghasemaghaei et al. (2017); Warth et al. (2011)
Augmented Analytics Adoption (AAA)	Use of a new generation of analytics technologies that employ AI to automate and augment the analytics lifecycle.	Sallam et al. (2017); Prat (2019)
Firm Agility (FA)	Dynamic capability reflecting the ability to sense opportunities and rapidly redesign or adapt processes in response to market conditions.	Ashrafi et al. (2019); Ghasemaghaei et al. (2017)
Firm Competitive Advantage (FCA)	Superior business position or performance relative to rivals through effective deployment and use of valuable resources.	Peters et al. (2016)

### 3.2 Measurement specification of augmented analytics adoption

AAA is modeled as a formative rather than reflective construct because its four indicators capture complementary facets that together define the extent of adoption, rather than interchangeable expressions of one underlying trait—they need not correlate strongly and may respond to different antecedents and consequences. Dropping any one indicator would narrow the conceptual domain that AAA is meant to represent, unlike removing a redundant item from a reflective scale. Assessment of the formative measure therefore focuses on indicator collinearity, the size and significance of outer weights, outer loadings, and substantive relevance. Because the archived dataset did not include a global AAA criterion item, a redundancy analysis could not be reconstructed after the fact; this gap is noted as a measurement limitation

### 3.3 Population, sampling strategy, and sample size determination

In this study, the population includes professionals who meet the research eligibility criteria and can provide informed assessments of augmented analytics adoption and its organizational implications. The targeted population therefore comprises professionals who directly engage with business analytics activities or govern the supporting information systems and data resources, including data analysts, BI specialists, data engineers, AI/ML practitioners, analytics developers, and IT executives/managers across multiple Jordanian sectors.

Because respondents needed working knowledge of analytics, data, or information systems, the study adopted a purposive, non-probability sampling strategy. Eligibility was restricted to individuals in Jordan-based organizations holding operational or managerial roles that exposed them to analytics-related activities or the surrounding IS/IT environment. This raised the odds that participants could answer the survey meaningfully, though it comes at the cost of statistical representativeness across Jordanian organizations generally.

To avoid over-reliance on a single organizational context, the sampling effort deliberately pursued diversity across sectors and job roles, drawing in respondents with technical, analytical, managerial, and business-facing responsibilities. This breadth adds heterogeneity to the sample, though the purposive design still stops short of supporting probability-based claims of representativeness across Jordanian firms.

We first assessed sample-size adequacy using the 10-times rule together with a minimum-*R^2^* criterion, yielding minimum thresholds of 60 and 75 observations, respectively. Of the 307 questionnaire responses received, 287 cases survived data screening and preparation and formed the final analytic sample. *N* = 287 is used consistently throughout the revised manuscript—in the measurement model, structural model, mediation analysis, effect-size assessment, and common-method-bias diagnostic—so every reported result rests on the same dataset. This final sample comfortably exceeds the original minimum thresholds, although the purposive sampling approach still limits how far the findings can be generalized statistically. As an independent check, the archived SmartPLS report itself lists the analyzed data file as containing 287 records, confirming that the PLS-SEM estimates in this revision are indeed based on *N* = 287. The population, purposive sampling, and sample-size adequacy procedure is summarized in Algorithm 1.

Algorithm 1 Population definition, purposive sampling, and sample size adequacy for PLS-SEM.

**Table:** 

Input	Eligibility criteria *E*; sector set *S*; role set *R*; PLS-SEM model; maximum number of incoming structural paths; formative-indicator count; and the minimum-*R^2^* sample-size requirement.
Output	Final analytic dataset *D* with |*D*| = 287 and |*D*| ≥ Nmin.
Step 1	Define the target population *P* as respondents who satisfy the eligibility criteria and work in a Jordanian business context.
Step 2	Construct a purposive sampling frame across relevant sectors and roles.
Step 3	Screen and recruit respondents with direct exposure to analytics, BI, data, or the supporting IS/IT environment.
Step 4	Administer the questionnaire and collect the returned responses (Nreturned = 307).
Step 5	Calculate the 10-times-rule requirement using the maximum number of incoming paths and the formative-indicator count; the original threshold was 60.
Step 6	Set Nmin as the larger of the 10-times-rule threshold and the minimum-*R^2^* threshold; the original minimum-*R^2^* requirement was 75.
Step 7	Conduct data screening and preparation and retain the traceable final analytic dataset (*D* = 287 cases).
Step 8	Verify adequacy: 287 ≥ 75; proceed to measurement-model and structural-model assessment in PLS-SEM.

### 3.4 Data collection

Business research can use several data-collection approaches, including interviews, focus groups, observation, and secondary data. A structured questionnaire survey was selected for this study because it permits standardized measurement of multiple constructs across respondents and supports statistical examination of relationships within the proposed model. The combined paper and digital format also allowed the instrument to reach respondents across different organizations and sectors while using the same measurement items.

The questionnaire was distributed in both paper and digital form across several business sectors. Of the 500 copies distributed, 307 were returned (a response rate of 61.4%), and 287 cases remained after screening and preparation for the final analysis. Every descriptive, measurement-model, structural-model, mediation, effect-size, and diagnostic result reported in the revised manuscript is therefore based on this same final analytic sample (*N* = 287). Data collection continued over 2 months; participation was voluntary and uncompensated, and respondents were told their information would be used strictly for research purposes.

Potential respondents were reached through organizational and professional channels—company information and professional profiles among them—to increase the likelihood that participants had genuine exposure to analytics or BI work. These steps aimed to raise the relevance of the responses collected while still protecting voluntary participation and confidentiality (Table 2).

**Table 2:** Survey administration and response summary.

Item	Value	Notes/Interpretation
Survey modality	Paper + Digital	Mixed-mode distribution
Questionnaires distributed	500	Combined count across both modes
Questionnaires returned	307	Response rate = 61.4%
Final analytic sample	287	Cases retained after screening and data preparation
Returned responses not included in the final analytic dataset	20	307 returned minus 287 retained
Final analytic rate among returned surveys	93.5%	287/307 × 100
Data collection duration	2 months	Continuous administration and follow-up
Sampling channels	Company websites + LinkedIn	Identification and expertise screening
Incentives	None	Voluntary participation
Confidentiality assurance	Yes	Data used for research purposes

### 3.5 Common method Bias and reproducibility considerations

Because the predictor, mediator, and outcome variables all came from the same cross-sectional questionnaire, we checked common method bias (CMB) as an additional diagnostic. Full-collinearity VIF values computed from the latent-variable scores fell between 2.503 and 3.224—under the 3.3 guideline applied here (Kock, 2015). The test cannot rule out common method bias entirely, but it indicates no severe full-collinearity CMB in the model.

### 3.6 Data analysis

Structural Equation Modeling (SEM) allows simultaneous assessment of measurement and structural relationships among latent variables. PLS-SEM was chosen here given the model’s relative complexity, its mix of reflective and formative measurement, and its emphasis on explained variance and prediction (Hair et al., 2023; Fischer et al., 2023). Analysis followed the conventional two-stage sequence—measurement-model assessment first, then structural-model assessment—with revised statistics drawn from the same 287-case SmartPLS analysis, including the formative AAA construct and the specified mediation relationship.

SmartPLS 3.3.2 was used for the analysis, applying the path-weighting scheme. The archived bootstrapping run drew 500 bootstrap samples, applied two-tailed testing at *α* = 0.05, and used bias-corrected and accelerated (BCa) confidence intervals—so hypothesis decisions rest on the bootstrap confidence interval alongside the path coefficient, *t* statistic, and *p* value. The reported assessment covers reliability and validity of reflective constructs, formative-indicator assessment, structural collinearity, *R^2^*, *f^2^*, direct, indirect, and total effects, and mediation. The archived output is presented exactly as originally generated, with the number of bootstrap subsamples left unchanged from the original run.

To restore the predictive-relevance assessment from the original submission without mixing results from different historical runs, we first analyzed the archived 287-case standardized indicator matrix using the same PLS path-weighting specification. The reproduced path coefficients matched the archived estimates to numerical precision, confirming that the model setup was consistent. Cross-validated redundancy was then computed for the reflectively measured endogenous constructs using an omission distance of *D* = 8, chosen because 287/8 is non-integer and falls within the conventional blindfolding range. AAA was excluded from the *Q^2^* calculation because it is specified formatively.

For reproducibility, this revision avoids combining coefficients from different historical SmartPLS runs. All path coefficients, confidence intervals, measurement statistics, *R^2^* and *f^2^* values, and mediation estimates reported below come from one traceable 287-case analysis; any output that could not be traced back to that run is not reported as a revalidated statistic.

The archived SmartPLS report lists the analyzed data file as containing 287 records, and its latent-variable descriptives likewise report 287 observations; these archived outputs form the basis for all inferential results presented below (Table 3).

**Table 3:** Structural model assessment protocol.

Assessment component	Metric/Output	Interpretation
Structural collinearity	Inner VIF	All predictor VIFs are below 3.0; no critical structural collinearity
Direct effects	*β*, *t*, *p*, BCa 95% CI	Robust support when the BCa 95% CI excludes zero
Explanatory power	*R^2^*	Proportion of variance explained in AAA, FA, and FCA
Effect size	*f^2^*	Approximate interpretation: 0.02 small, 0.15 medium, 0.35 large
Mediation	Direct, specific indirect, total effects + BCa CI	Indirect effect supported when its BCa interval excludes zero
Common method bias diagnostic	Full-collinearity VIF	Values ≤3.3 suggest severe CMB is unlikely under this diagnostic (Kock, 2015)
Predictive relevance	*Q^2^* (cross-validated redundancy)	Blindfolding with *D* = 8; *Q^2^* > 0 indicates predictive relevance for reflective endogenous constructs

### 3.7 Structural model specification

After assessing the measurement model, the structural (inner) model evaluates the hypothesized relationships among the latent constructs. This stage examines predictor collinearity, the size and statistical uncertainty of path coefficients, variance explained in the endogenous constructs, effect sizes, and mediation, using non-parametric bootstrapping to estimate the sampling distribution of path coefficients and indirect effects.

Let AAA, FA, and FCA denote Augmented Analytics Adoption, Firm Agility, and Firm Competitive Advantage, respectively. The structural model tested in this study is specified as:

AAA = β₁IT-INF + β₂DTF + β₃ITF + β₄TTF + β₅CTF + β₆ATF + ζAAA

FA = β(H7a)AAA + ζFA

FCA = β(H7c)AAA + β(H7b)FA + ζFCA

where *β* denotes a standardized structural path coefficient, its subscript identifies the corresponding hypothesis, and ζ denotes the residual term for each endogenous construct. The mediation mechanism is evaluated through the indirect effect AAA → FA → FCA together with the direct AAA → FCA effect.

## 4 Results

### 4.1 Respondent profile

The final analytic sample comprised 287 respondents drawn from Jordan-based organizations, spanning a range of industries, job roles, educational backgrounds, experience levels, and firm sizes. The demographic profile reported here is restricted to this same *N* = 287, aligning with the sample used throughout the PLS-SEM results.

Of the 287 respondents, 191 (66.6%) were male, and 96 (33.4%) were female. In terms of education, 151 respondents (52.6%) held a bachelor’s degree, 115 (40.1%) held a master’s degree, 15 (5.2%) held a PhD, and 6 (2.1%) reported a two-year college qualification. The two largest age groups were 20–29 years and 30–39 years, each with 121 respondents (42.2%); 44 respondents (15.3%) were aged 40–49, and one respondent (0.3%) was aged 50 or above.

The industry profile was diverse, with services and education each accounting for 48 respondents (16.7%), followed by communication and finance with 34 respondents each (11.8%) and retail with 29 respondents (10.1%). The grouped job-role profile included managers and executives (23.0%), business users (20.2%), data scientists and engineers (17.4%), BI and AI developers (16.0%), data and business analysts (15.3%), and BI/analytics education and training roles (8.0%). With respect to experience, 29.3% reported 6–10 years, 28.2% reported 2–5 years, 22.6% reported more than 10 years, and 19.9% reported under 2 years. Firm size was also distributed across categories: 35.2% worked in firms with fewer than 50 employees, 36.9% in firms with 50–500 employees, and 27.9% in firms with more than 500 employees (Figure 2; Table 4).

**Figure 2:**
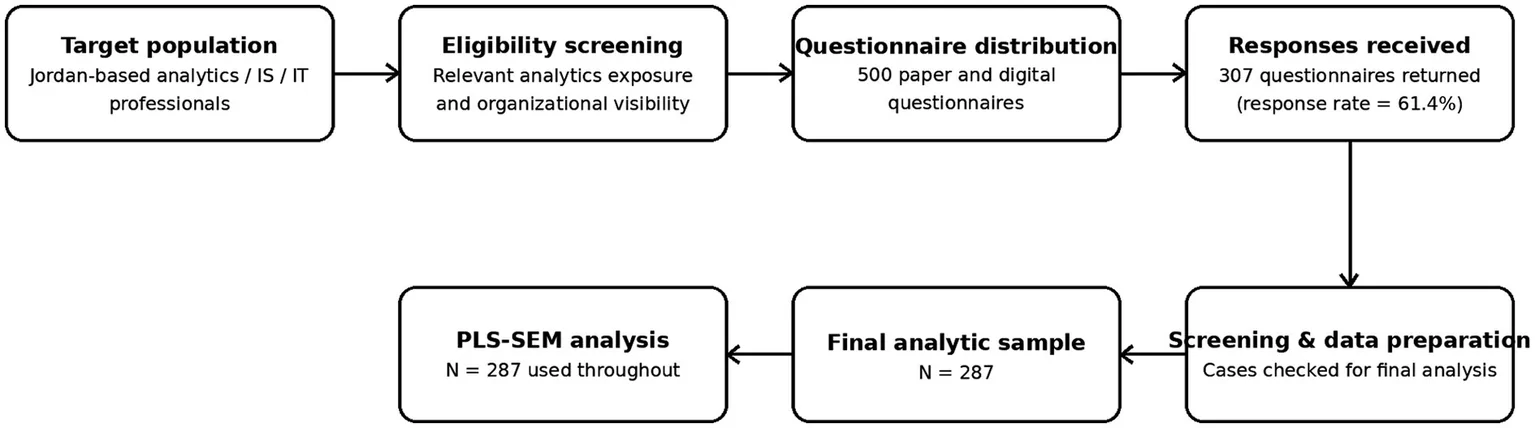
Sampling and final analytic sample workflow (*N* = 287). This figure lays out the sampling and sample-size workflow from start to finish—beginning with the eligibility criteria and purposive sampling frame, moving through questionnaire administration and data screening, and concluding with confirmation that the final analytic dataset meets the minimum sample-size requirement before measurement and structural model estimation begin.

**Table 4:** Demographic profile of the final analytic sample (*N* = 287).

Demographic	Category	Frequency	Percentage
Gender	Male	191	66.6%
	Female	96	33.4%
Education	College (2 years)	6	2.1%
	Bachelor (4 years)	151	52.6%
	Master’s degree	115	40.1%
	PhD	15	5.2%
Age	20–29	121	42.2%
	30–39	121	42.2%
	40–49	44	15.3%
	50+	1	0.3%
Industry	Services	48	16.7%
	Finance	34	11.8%
	Education	48	16.7%
	Manufacturing	13	4.5%
	Healthcare	20	7.0%
	Energy and utilities	7	2.4%
	Retail	29	10.1%
	Media	4	1.4%
	Transportation	12	4.2%
	Communication	34	11.8%
	Construction	7	2.4%
	Government or NGOs	21	7.3%
	Others	10	3.5%
Job role	Manager & executive	66	23.0%
	Data & business analyst	44	15.3%
	BI&A education and training	23	8.0%
	BI & AI developer	46	16.0%
	Data scientist & engineer	50	17.4%
	Business users	58	20.2%
Work experience	Under 2 years	57	19.9%
	2–5 years	81	28.2%
	6–10 years	84	29.3%
	Above 10 years	65	22.6%
Number of employees in the firm	<50 employees	101	35.2%
	50–500 employees	106	36.9%
	>500 employees	80	27.9%

This spread offers useful heterogeneity for testing the proposed relationships across different organizational settings. Because sampling was purposive rather than probability-based, however, this demographic diversity should not be read as evidence that the sample represents the Jordanian business population as a whole (Figure 3).

**Figure 3:**
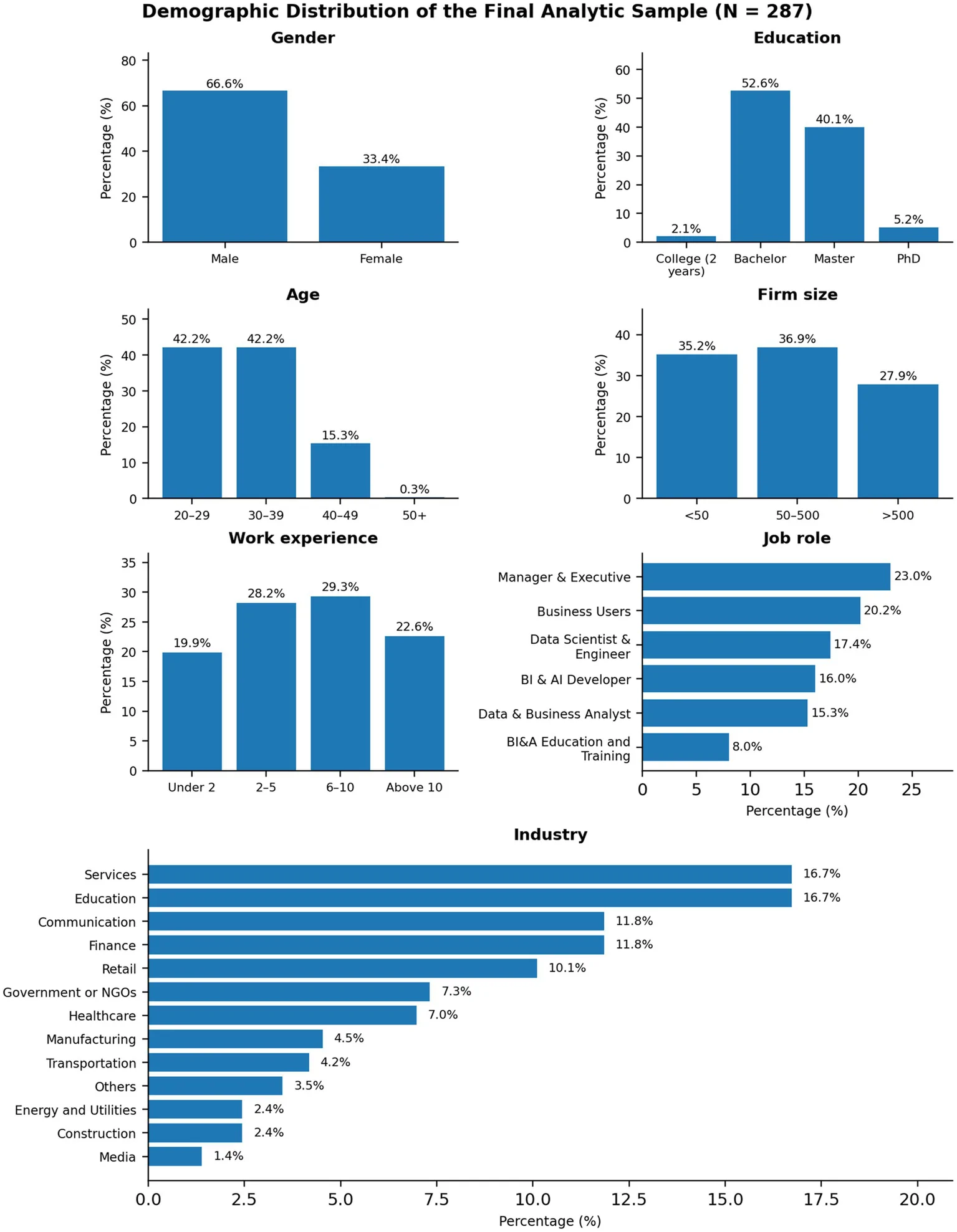
Demographic distribution of the final analytic sample (*N* = 287). Bars indicate percentage share per category; industry and grouped job-role variables are presented as horizontal bar charts for readability.

### 4.2 Reflective measurement model

Assessment of the reflective measurement model focuses on whether the indicators measure their intended constructs with adequate reliability and validity. The evaluation therefore considers indicator loadings and Average Variance Extracted (AVE) for convergent validity; cross-loadings and the Fornell–Larcker criterion for discriminant validity; the HTMT ratio as an additional discriminant-validity diagnostic; and Cronbach’s alpha and Composite Reliability for internal consistency (Hair et al., 2023; Hair et al., 2011; Guenther et al., 2023; Fischer et al., 2023; Hair et al., 2019).

#### 4.2.1 Convergent validity

Convergent validity rested on indicator loadings and Average Variance Extracted (AVE), where loadings near 0.70 or above and AVE values above 0.50 are generally taken as acceptable for reflective constructs (Hair et al., 2023; Fischer et al., 2023; Hair et al., 2019). Reflective loadings in the 287-case analysis span 0.703 to 0.861, with AVE values between 0.588 and 0.691—results that support convergent validity across the reflective constructs (Table 5).

**Table 5:** Reflective constructs, indicator loadings, and AVE.

Construct	Item	Outer loading	AVE
IT-INF	INF1	0.800	0.598
	INF2	0.781	
	INF3	0.835	
	INF4	0.729	
	INF5	0.716	
DTF	DTF1	0.757	0.612
	DTF2	0.801	
	DTF3	0.789	
	DTF4	0.780	
ITF	ITF1	0.796	0.655
	ITF2	0.814	
	ITF3	0.823	
	ITF4	0.806	
TTF	TTF1	0.830	0.691
	TTF2	0.802	
	TTF3	0.861	
CTF	CTF1	0.795	0.684
	CTF2	0.847	
	CTF3	0.838	
ATF	ATF1	0.790	0.688
	ATF2	0.853	
	ATF3	0.835	
	ATF4	0.839	
FA	FA1	0.749	0.588
	FA2	0.744	
	FA3	0.703	
	FA4	0.818	
	FA5	0.805	
	FA6	0.788	
	FA7	0.753	
FCA	FCA1	0.732	0.629
	FCA2	0.763	
	FCA3	0.830	
	FCA4	0.815	
	FCA5	0.816	
	FCA6	0.796	

#### 4.2.2 Discriminant validity

Discriminant validity was checked through cross-loadings, the Fornell-Larcker criterion, and the Heterotrait-Monotrait (HTMT) ratio. The Fornell-Larcker results hold up well. Most HTMT values stay below 0.90, though ITF-TTF reaches 0.902, and DTF-ITF and DTF-TTF sit close to that mark; these particular pairs are therefore read cautiously rather than treated as unequivocal evidence of discriminant validity.

Cross-loadings offer an item-level check on discriminant validity by comparing each indicator’s loading on its assigned construct with its loadings on other constructs. For reflective indicators, the loading on the intended construct should, as a rule, exceed the cross-loadings on competing constructs (Table 6).

**Table 6:** Cross-loadings (AAA entries reported as diagnostic only).

Item	AAA	ATF	CTF	DTF	FA	FCA	IT-INF	ITF	TTF
AAA1	0.812	0.543	0.523	0.538	0.494	0.432	0.576	0.555	0.507
AAA2	0.782	0.492	0.499	0.478	0.492	0.445	0.496	0.520	0.501
AAA3	0.767	0.523	0.515	0.540	0.495	0.389	0.448	0.500	0.483
AAA4	0.781	0.526	0.514	0.534	0.487	0.433	0.472	0.480	0.443
ATF1	0.504	0.790	0.467	0.458	0.450	0.362	0.437	0.526	0.544
ATF2	0.580	0.853	0.618	0.581	0.554	0.520	0.592	0.583	0.574
ATF3	0.548	0.835	0.594	0.548	0.564	0.464	0.536	0.589	0.551
ATF4	0.565	0.839	0.596	0.502	0.539	0.491	0.588	0.545	0.559
CTF1	0.475	0.522	0.795	0.495	0.490	0.443	0.522	0.479	0.507
CTF2	0.572	0.594	0.847	0.574	0.532	0.500	0.574	0.566	0.552
CTF3	0.563	0.587	0.838	0.639	0.482	0.434	0.586	0.594	0.572
DTF1	0.549	0.483	0.508	0.757	0.476	0.394	0.496	0.536	0.513
DTF2	0.510	0.494	0.500	0.801	0.406	0.421	0.502	0.563	0.559
DTF3	0.500	0.494	0.571	0.789	0.389	0.351	0.564	0.598	0.570
DTF4	0.518	0.502	0.587	0.780	0.504	0.444	0.582	0.555	0.548
FA1	0.590	0.540	0.544	0.494	0.749	0.552	0.492	0.504	0.556
FA2	0.466	0.479	0.538	0.476	0.744	0.523	0.427	0.430	0.532
FA3	0.443	0.392	0.405	0.356	0.703	0.502	0.369	0.352	0.417
FA4	0.516	0.522	0.481	0.436	0.818	0.609	0.488	0.412	0.473
FA5	0.479	0.583	0.499	0.442	0.805	0.616	0.507	0.437	0.490
FA6	0.430	0.456	0.466	0.417	0.788	0.654	0.472	0.410	0.413
FA7	0.426	0.433	0.323	0.430	0.753	0.676	0.381	0.349	0.383
FCA1	0.382	0.410	0.449	0.344	0.568	0.732	0.363	0.394	0.359
FCA2	0.453	0.465	0.388	0.437	0.587	0.763	0.401	0.349	0.353
FCA3	0.509	0.485	0.476	0.437	0.614	0.830	0.452	0.446	0.474
FCA4	0.407	0.423	0.421	0.389	0.645	0.815	0.493	0.400	0.417
FCA5	0.380	0.429	0.468	0.449	0.607	0.816	0.444	0.408	0.442
FCA6	0.444	0.437	0.442	0.395	0.648	0.796	0.419	0.413	0.469
INF1	0.529	0.536	0.582	0.542	0.438	0.394	0.800	0.493	0.464
INF2	0.510	0.492	0.555	0.582	0.493	0.417	0.781	0.540	0.484
INF3	0.520	0.545	0.563	0.569	0.533	0.479	0.835	0.485	0.509
INF4	0.448	0.494	0.460	0.471	0.410	0.365	0.729	0.459	0.432
INF5	0.441	0.450	0.453	0.476	0.382	0.443	0.716	0.442	0.361
ITF1	0.518	0.612	0.615	0.624	0.524	0.455	0.565	0.796	0.657
ITF2	0.505	0.512	0.524	0.571	0.406	0.441	0.506	0.814	0.565
ITF3	0.549	0.525	0.449	0.557	0.373	0.366	0.425	0.823	0.520
ITF4	0.541	0.541	0.566	0.579	0.447	0.385	0.536	0.806	0.603
TTF1	0.528	0.484	0.487	0.556	0.468	0.391	0.496	0.582	0.830
TTF2	0.436	0.569	0.528	0.537	0.506	0.429	0.444	0.568	0.802
TTF3	0.553	0.625	0.625	0.645	0.543	0.501	0.514	0.650	0.861

The Fornell–Larcker criterion sets the square root of each reflective construct’s AVE against its correlations with other constructs, on the logic that a construct should share more variance with its own indicators than with others in the model. The revalidated 287-case results satisfy this criterion for the reflective constructs (Table 7).

**Table 7:** Fornell-Larcker criterion for reflective constructs.

Construct	ATF	CTF	DTF	FA	FCA	IT-INF	ITF	TTF
ATF	0.830	–	–	–	–	–	–	–
CTF	0.688	0.827	–	–	–	–	–	–
DTF	0.631	0.692	0.782	–	–	–	–	–
FA	0.637	0.606	0.569	0.767	–	–	–	–
FCA	0.557	0.555	0.515	0.773	0.793	–	–	–
IT-INF	0.652	0.679	0.685	0.586	0.542	0.773	–	–
ITF	0.676	0.664	0.720	0.540	0.507	0.627	0.810	–
TTF	0.672	0.659	0.700	0.608	0.530	0.585	0.724	0.831

HTMT offers a more sensitive read on discriminant validity, weighing between-construct indicator correlations against within-construct correlations; values at or above 0.90 caLL for scrutiny when the constructs involved are conceptually related. Here, the ITF–TTF value of 0.902 and the near-90 DTF–ITF and DTF–TTF values are accordingly read with caution rather than taken as unequivocal evidence of separation (Table 8).

**Table 8:** Heterotrait-Monotrait ratio (HTMT).

Construct	ATF	CTF	DTF	FA	FCA	IT-INF	ITF	TTF
ATF	–	–	–	–	–	–	–	–
CTF	0.846	–	–	–	–	–	–	–
DTF	0.769	0.884	–	–	–	–	–	–
FA	0.731	0.736	0.680	–	–	–	–	–
FCA	0.641	0.675	0.618	0.873	–	–	–	–
IT-INF	0.772	0.844	0.845	0.680	0.633	–	–	–
ITF	0.808	0.831	0.893	0.633	0.596	0.758	–	–
TTF	0.827	0.847	0.890	0.735	0.637	0.721	0.902	–

#### 4.2.3 Reliability

Reliability speaks to how consistently a set of reflective indicators measures its construct. Cronbach’s alpha captures inter-item consistency, while Composite Reliability factors in the estimated indicator loadings; values around 0.70 or higher are generally judged satisfactory (Hair et al., 2023; Hair et al., 2011). In the 287-case analysis, composite reliability ranges from 0.863 to 0.910 and Cronbach’s alpha from 0.769 to 0.883, indicating satisfactory internal consistency (Table 9).

**Table 9:** Composite reliability and Cronbach’s alpha.

Construct	Composite reliability	Cronbach’s alpha
ATF	0.898	0.849
CTF	0.866	0.769
DTF	0.863	0.788
FA	0.909	0.883
FCA	0.910	0.881
IT-INF	0.881	0.831
ITF	0.884	0.825
TTF	0.870	0.778

### 4.3 Formative measurement model for AAA

Formative indicators call for a different evaluation logic than reflective ones, since they jointly define a construct rather than reflect a single underlying trait. Here, indicator relevance is judged from the size and significance of outer weights, collinearity is checked via VIF, and outer loadings are considered alongside these when deciding whether an indicator contributes meaningfully to the construct (Leguina, 2015; Hair et al., 2023).

For the formative AAA construct, the assessment centered on outer weights, their statistical significance, and indicator collinearity. The four outer weights fall between 0.268 and 0.367, all statistically significant; outer loadings range from 0.767 to 0.812; and VIF values sit between 1.458 and 1.706—together supporting retention of all four formative indicators (Figure 4; Table 10).

**Figure 4:**
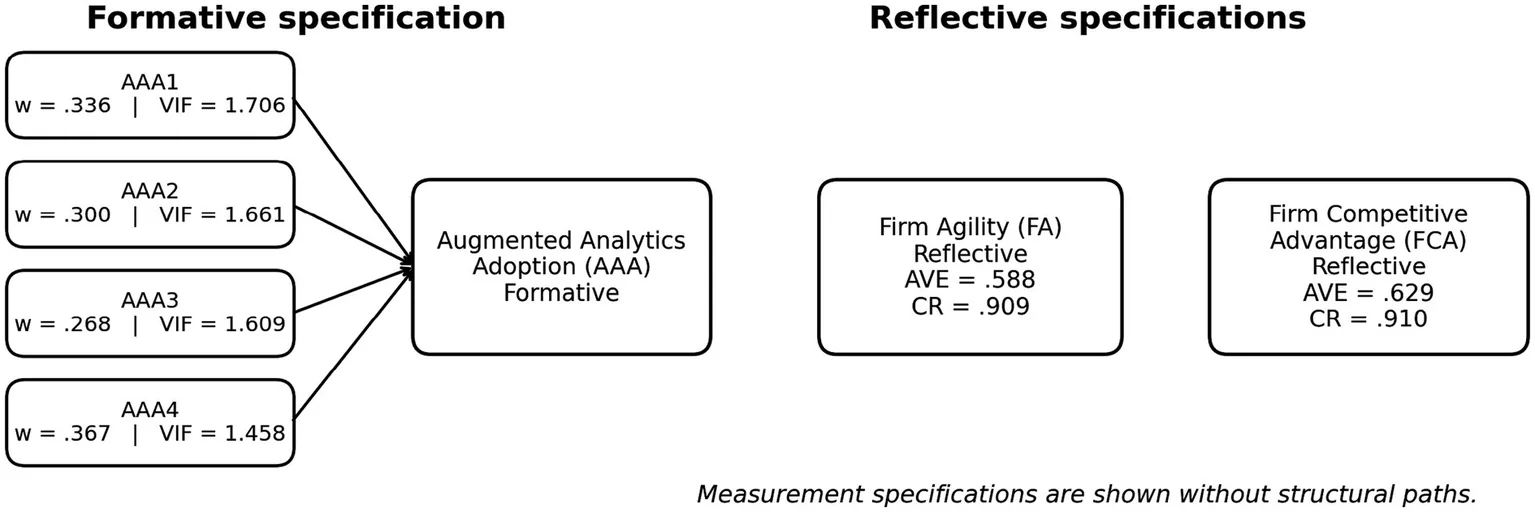
Measurement-model summary.

**Table 10:** AAA formative outer weights, outer loadings, significance, and VIF.

Measure	Outer weight (outer loading)	*p*-value	VIF
AAA1	0.336 (0.812)	<0.001	1.706
AAA2	0.300 (0.782)	<0.001	1.661
AAA3	0.268 (0.767)	=0.001	1.609
AAA4	0.367 (0.781)	<0.001	1.458

### 4.4 Structural model

After the measurement model was satisfactorily assessed, the structural model was examined to evaluate the proposed relationships among constructs. Standardized path coefficients indicate the direction and magnitude of the relationships, while bootstrapping provides *t*-statistics, *p*-values, and confidence intervals for inference. Structural VIF values are examined before interpreting the paths so that problematic predictor collinearity does not distort the estimates.

The structural model was assessed after the measurement model. The evaluation covered predictor collinearity, path coefficients, *R^2^*, *f^2^*, and mediation. Inner VIF values for the six predictors of AAA range from 2.400 to 2.939. The VIF for AAA predicting FA is 1.000, and the two predictors of FCA each have a VIF of 1.641. These values do not indicate critical structural collinearity.

#### 4.4.1 Structural model path coefficients

Standardized path coefficients can range from −1 to +1, with larger absolute values signaling stronger relationships. Support for a given path was determined from the BCa bootstrap confidence interval, read together with the coefficient, t statistic, and *p* value—a path counted as supported only when its confidence interval excluded zero. This distinction matters most for H1 and H7c, where both paths carry *p*-values below 0.05 yet their BCa 95% confidence intervals edge just past zero. Both are accordingly classified as not supported under the confidence-interval rule, which avoids the contradictions that can arise from relying on *p*-values alone (Table 11).

**Table 11:** Hypothesis testing and direct structural paths.

Hypothesis	Relationship	*β*	*t* (*p*)	BCa 95% CI	Decision
H1	IT-INF → AAA	0.146	2.133 (=0.033)	[−0.002, 0.266]	Not supported under BCa CI
H2	DTF → AAA	0.188	2.603 (=0.009)	[0.058, 0.339]	Supported
H3	ITF → AAA	0.150	2.001 (=0.046)	[0.012, 0.309]	Supported
H4	TTF → AAA	0.047	0.728 (=0.467)	[−0.100, 0.163]	Not supported
H5	CTF → AAA	0.141	2.111 (=0.035)	[0.014, 0.270]	Supported
H6	ATF → AAA	0.219	3.000 (=0.003)	[0.077, 0.352]	Supported
H7a	AAA → FA	0.625	14.539 (<0.001)	[0.533, 0.697]	Supported
H7b	FA → FCA	0.712	18.207 (<0.001)	[0.636, 0.791]	Supported
H7c	AAA → FCA	0.097	2.108 (=0.036)	[−0.005, 0.179]	Not supported under BCa CI

Not all six fit dimensions relate to AAA in the same way, as the direct-path results make clear. Analytics-technology fit, data-technology fit, individual-technology fit, and culture-technology fit each show positive effects with BCa intervals excluding zero. IT infrastructure-technology fit is positive, but its interval includes zero, and task-technology fit is small and non-significant. Downstream, AAA relates strongly to FA, and FA relates strongly to FCA, while the direct AAA-FCA coefficient is positive but small, with an interval that includes zero.

#### 4.4.2 Mediation analysis

Mediation describes a situation in which an independent variable’s effect on an outcome passes through an intervening variable. Testing for it here means examining the AAA → FA path, the FA → FCA path, the direct AAA → FCA effect, and the specific indirect AAA → FA → FCA effect, with bootstrapped confidence intervals used to judge whether the indirect effect excludes zero (Preacher and Hayes, 2008; Nitzl et al., 2016).

AAA’s specific indirect effect on FCA through FA is positive, and its BCa confidence interval excludes zero, while the direct AAA-FCA effect fails to clear the BCa interval threshold. Together, these results point to indirect-only mediation (Preacher and Hayes, 2008; Nitzl et al., 2016; Zhao et al., 2010): in practical terms, augmented analytics adoption appears linked to competitive advantage mainly through firm agility (Figure 5; Table 12).

**Figure 5:**
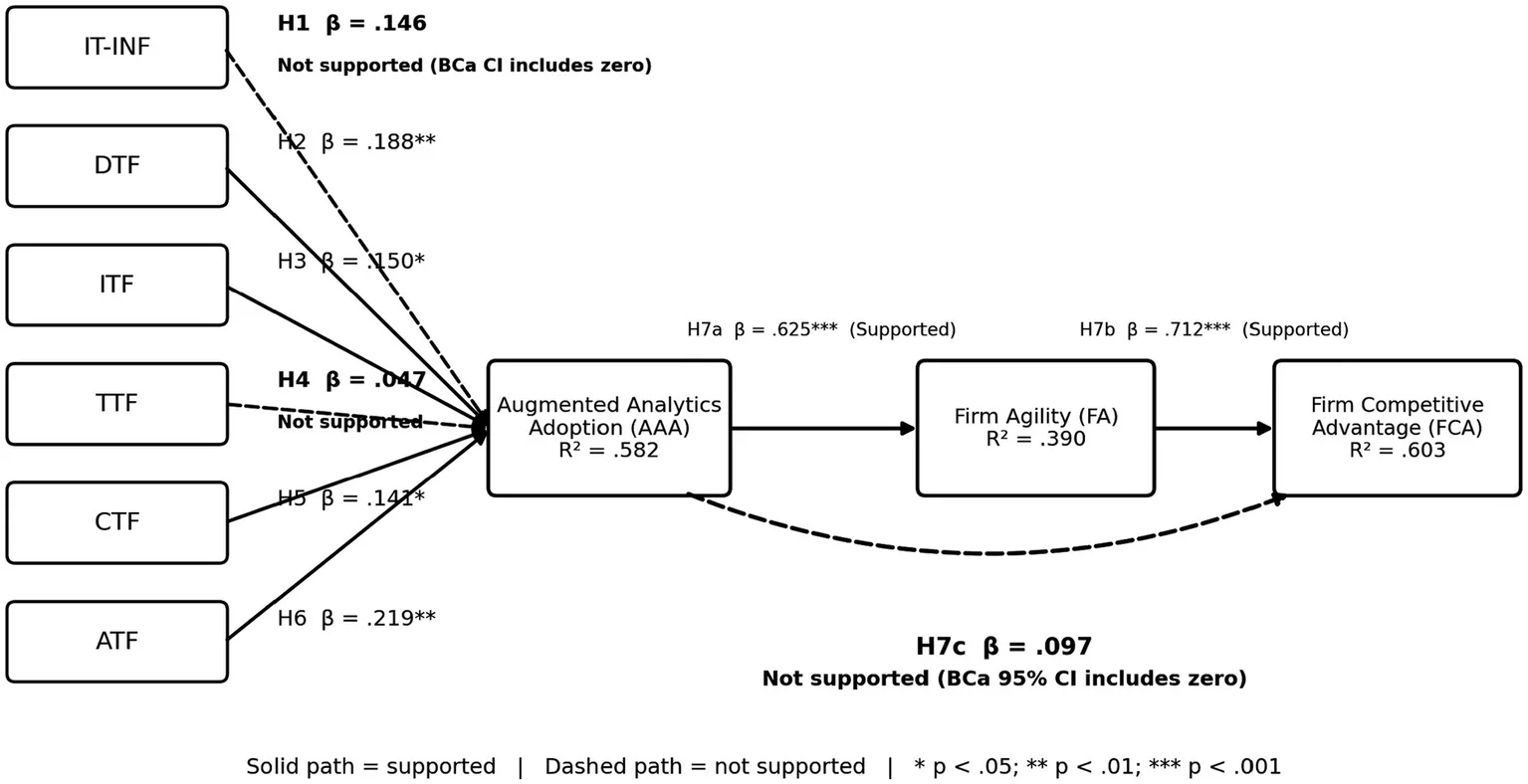
Structural model results.

**Table 12:** Decomposition of the AAA–FA–FCA mediation relationship.

Effect	Relationship	*β*	*t* (*p*)	BCa 95% CI	Interpretation
Component path	AAA → FA	0.625	14.539 (<0.001)	[0.533, 0.697]	Robust positive path
Component path	FA → FCA	0.712	18.207 (<0.001)	[0.636, 0.791]	Robust positive path
Direct effect (c′)	AAA → FCA	0.097	2.108 (=0.036)	[−0.005, 0.179]	Positive but not robust under BCa CI
Specific indirect effect	AAA → FA → FCA	0.445	11.580 (<0.001)	[0.373, 0.521]	Significant; H8 supported
Total effect	AAA → FCA	0.542	11.751 (<0.001)	[0.443, 0.628]	Significant

#### 4.4.3 Coefficient of determination (*R^2^*)

*R^2^* captures the share of variance explained in each endogenous construct (Hair et al., 2019; Hair et al., 2013): the model accounts for 58.2% of the variance in AAA (*R^2^* = 0.582), 39.0% in FA (*R^2^* = 0.390), and 60.3% in FCA (*R^2^* = 0.603; Figure 6; Table 13).

**Figure 6:**
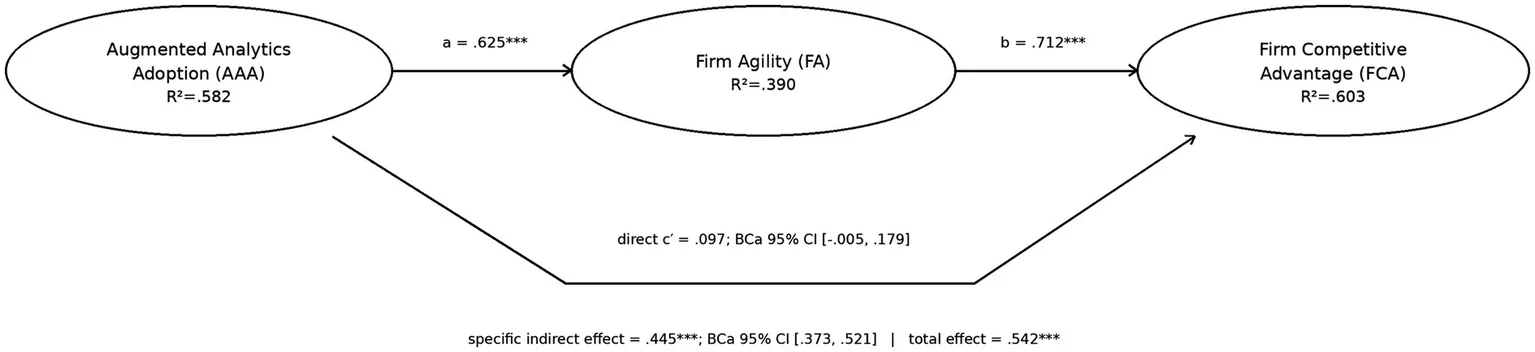
AAA–FA–FCA mediation pathway with BCa confidence intervals.

**Table 13:** *R^2^* Values of endogenous constructs.

Construct	*R^2^*	Interpretation
Augmented Analytics Adoption (AAA)	0.582	58.2% of variance explained
Firm Agility (FA)	0.390	39.0% of variance explained
Firm Competitive Advantage (FCA)	0.603	60.3% of variance explained

#### 4.4.4 Effect size (*f^2^*)

Effect size *f^2^* tracks how much an endogenous construct’s *R^2^* shifts when a given predictor is dropped, with values around 0.02, 0.15, and 0.35 conventionally read as small, medium, and large (Hair et al., 2023). The largest effects here belong to AAA on FA (*f^2^* = 0.641) and FA on FCA (*f^2^* = 0.779), while the direct AAA-FCA effect is negligible (*f^2^* = 0.014). Among the antecedents of AAA, ATF (*f^2^* = 0.045) and DTF (*f^2^* = 0.029) carry the largest incremental effects, though both remain small (Table 14).

**Table 14:** Effect size (*f^2^*) values.

Relationship	*f^2^*	Interpretation
AAA → FA	0.641	Large
AAA → FCA	0.014	Negligible
ATF → AAA	0.045	Small
CTF → AAA	0.018	Negligible
DTF → AAA	0.029	Small
FA → FCA	0.779	Large
IT-INF → AAA	0.021	Small
ITF → AAA	0.019	Negligible
TTF → AAA	0.002	Negligible

#### 4.4.5 Predictive relevance (*Q^2^*)

Predictive relevance was gauged using Stone–Geisser’s *Q^2^*, via blindfolding and the cross-validated redundancy approach (Hair et al., 2023; Hair et al., 2019), applied to the same 287-case indicator matrix and PLS specification used in the revalidated structural model. For the two reflectively measured endogenous constructs, *Q^2^* = 1 − SSE/SSO was computed from the sum of squared observations (SSO) and sum of squared prediction errors (SSE). At an omission distance of *D* = 8, *Q^2^* came out to 0.224 for Firm Agility (FA) and 0.374 for Firm Competitive Advantage (FCA)—both above zero, indicating predictive relevance, and notably stronger for FCA. AAA does not appear in the blindfolding table, since it is specified formatively rather than reflectively (Figure 7; Table 15).

**Figure 7:**
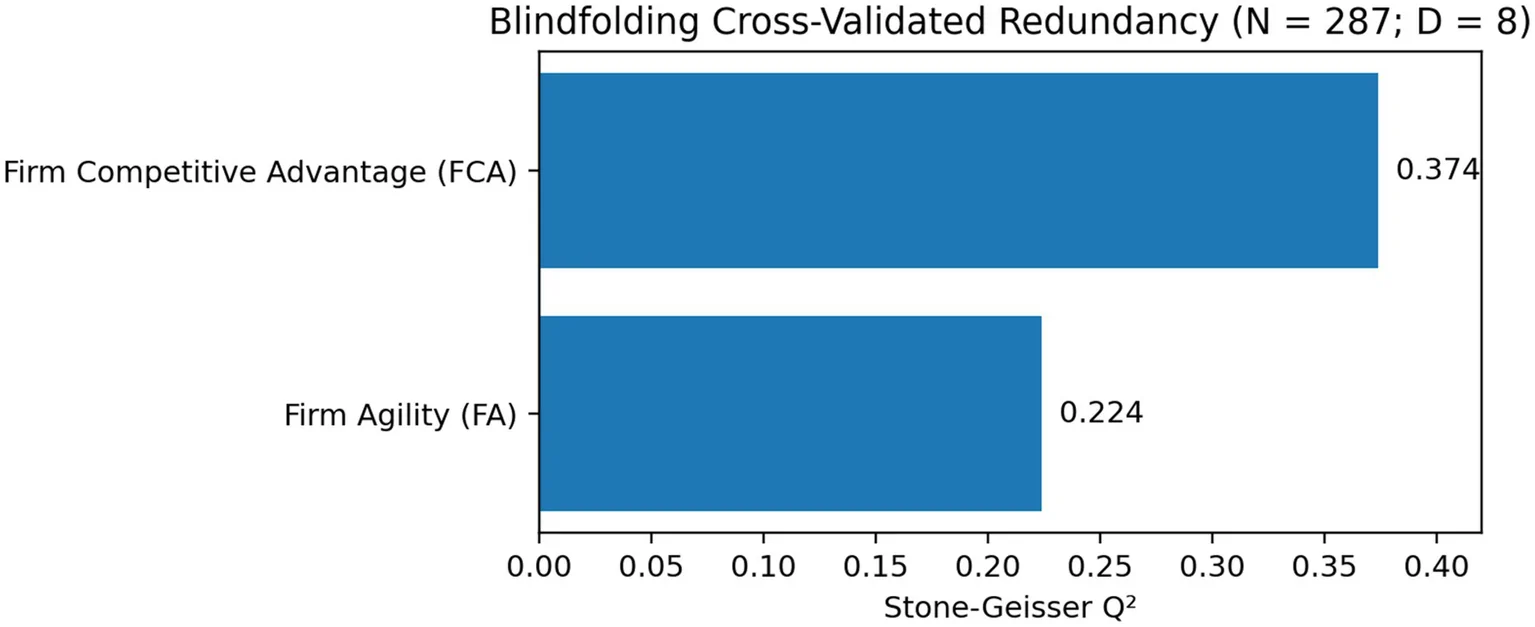
Blindfolding cross-validated redundancy results for the reflective endogenous constructs (*N* = 287; *D* = 8).

**Table 15:** Predictive relevance (*Q^2^*) of reflective endogenous constructs (*N* = 287; *D* = 8).

Construct	SSO	SSE	*Q^2^* (=1 − SSE/SSO)
Firm Agility (FA)	2009.000	1559.055	0.224
Firm Competitive Advantage (FCA)	1722.000	1077.788	0.374

#### 4.4.6 Common method Bias diagnostic

As a further check on common method bias, full-collinearity VIFs were computed from the SmartPLS latent-variable scores (Kock, 2015); values ranged from 2.503 to 3.224, comfortably under the 3.3 guideline for every construct. This does not eliminate the possibility of common method bias outright. Still, it offers no sign of severe full-collinearity CMB; however, reliance on a single self-report survey remains a limitation of the study (Figure 8; Table 16).

**Figure 8:**
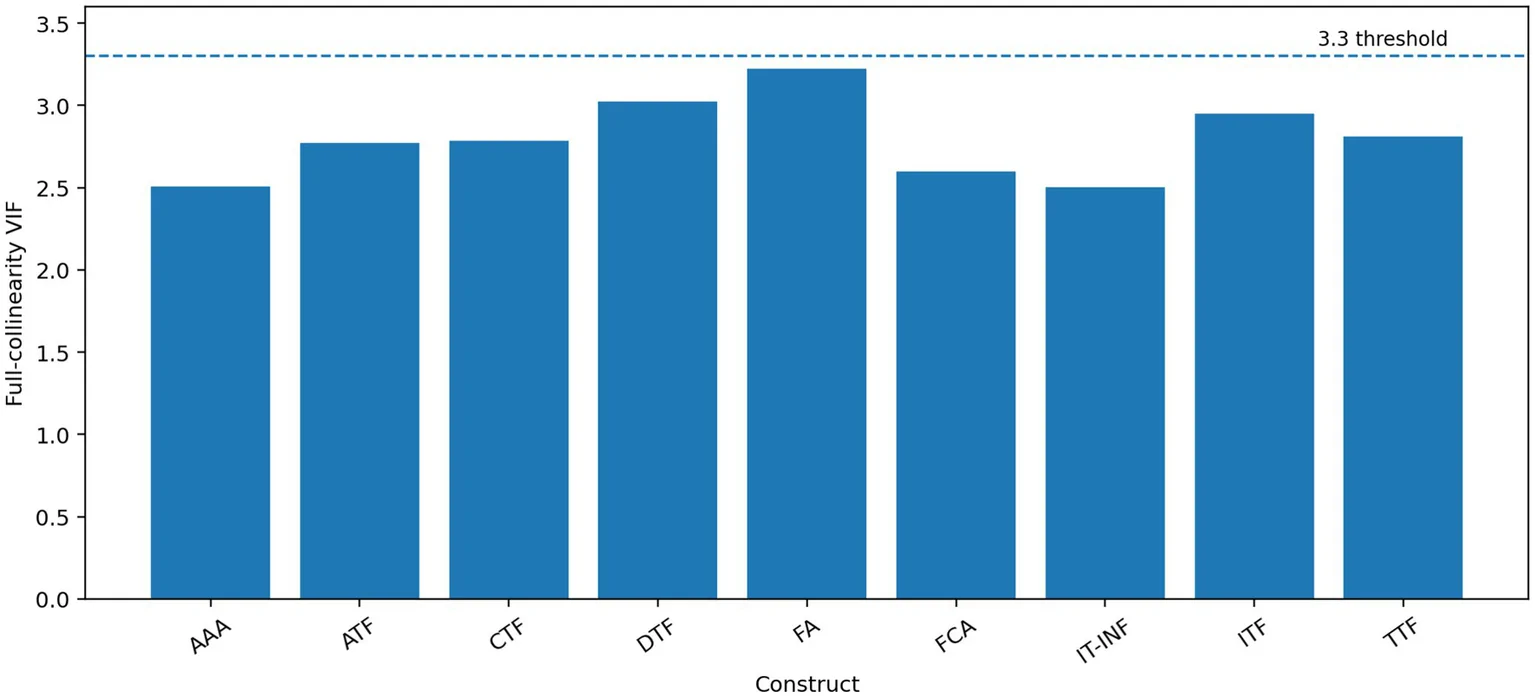
Full-collinearity VIF assessment for common method bias.

**Table 16:** Full-collinearity VIF assessment for common method bias.

Construct	Full-collinearity VIF	Threshold	Assessment
AAA	2.509	3.3	Below threshold
ATF	2.772	3.3	Below threshold
CTF	2.786	3.3	Below threshold
DTF	3.024	3.3	Below threshold
FA	3.224	3.3	Below threshold
FCA	2.599	3.3	Below threshold
IT-INF	2.503	3.3	Below threshold
ITF	2.948	3.3	Below threshold
TTF	2.810	3.3	Below threshold

## 5 Discussion

### 5.1 Key findings and interpretation

This study examined six resource-technology fit dimensions linked to augmented analytics adoption, then tested whether that adoption relates to competitive advantage both directly and indirectly through firm agility. The results showed a selective, rather than uniform, pattern: data-technology fit, individual-technology fit, culture-technology fit, and analytics capability-technology fit all held up, while task-technology fit and the IT-infrastructure path did not survive the BCa confidence-interval rule. This selectivity carries theoretical weight—it suggests that augmented analytics adoption hinges less on the mere presence of technology and more on whether data, people, analytical capability, and organizational norms are aligned with its use (Prat, 2019; Arunachalam et al., 2018; Côrte-Real et al., 2017; Muslimin et al., 2017; Ashrafi et al., 2019; Ammenwerth et al., 2006; Ghasemaghaei et al., 2017; Daradkeh, 2019; Lee et al., 2007; Warth et al., 2011; Gupta and George, 2016; Grover et al., 2018; Alghamdi and Al-Baity, 2022; Oesterreich et al., 2021).

The relationship between AAA and firm agility proved strong and positive (*β* = 0.625), echoing prior work showing that analytics capabilities sharpen an organization’s ability to sense change, process information, and respond quickly (Ashrafi et al., 2019; Ghasemaghaei et al., 2017; Atienza-Barba et al., 2024; Xu et al., 2024; Hyun et al., 2023). Augmented analytics can compress the path from data preparation to insight generation. Yet that speed matters only once decision rights, coordination, and operational processes allow the firm to act on it. This supports a dynamic-capability reading of the result: AAA functions as an information-processing resource. At the same time, agility is the higher-order capability that turns this resource into timely reconfiguration and response (Abdul Wahab and Radmehr, 2024; Mariani and Mancini, 2025; Sadeghi R et al., 2024; Alghamdi and Al-Baity, 2022; Oesterreich et al., 2021; Salazar and Kunc, 2025; Hyun et al., 2023).

The picture for competitive advantage is more nuanced. FA relates strongly to FCA (*β* = 0.712; *f^2^* = 0.779), but the direct AAA-FCA relationship is small (*β* = 0.097; *f^2^* = 0.014), and its BCa 95% confidence interval spans zero. This is not evidence that augmented analytics lacks strategic value—rather, it undercuts the assumption that adoption alone automatically yields advantage. Analytics resources generate value once embedded in business processes and paired with complementary organizational capabilities (Peters et al., 2016; Grover et al., 2018; Seddon et al., 2017; Wamba et al., 2015); here, agility is the capability that lets firms convert analytical insight into faster decisions, process adaptation, and market response, a reading consistent with recent AI-value and analytics-agility research (Atienza-Barba et al., 2024; Xu et al., 2024; Abdul Wahab and Radmehr, 2024; Mariani and Mancini, 2025; Sadeghi R et al., 2024; Salazar and Kunc, 2025; Hyun et al., 2023).

The indirect AAA-FA-FCA effect stands at *β* = 0.445 (BCa 95% CI [0.373, 0.521]), against a total AAA-FCA effect of *β* = 0.542. Because the indirect effect is supported while the direct effect fails the BCa interval rule, the pattern points to indirect-only mediation (Preacher and Hayes, 2008; Nitzl et al., 2016; Zhao et al., 2010). This ranks among the study’s strongest findings, since it uncovers an actual mechanism of value realization rather than simply reporting an association between adoption and performance—linking the fit-based antecedent model to the dynamic-capability argument: aligned resources facilitate adoption, and agility is what carries that adoption forward into competitive advantage (Ashrafi et al., 2019; Ghasemaghaei et al., 2017; Atienza-Barba et al., 2024; Xu et al., 2024; Abdul Wahab and Radmehr, 2024; Mariani and Mancini, 2025; Sadeghi R et al., 2024; Hyun et al., 2023).

These downstream results also line up in magnitude and direction with recent empirical work. Mariani and Mancini (2025) found that AI capabilities positively shaped organizational agility (*β* = 0.313, *p* < 0.001), which in turn positively affected value definition (*β* = 0.436, *p* < 0.001), value creation (*β* = 0.445, *p* < 0.001), and value capture (*β* = 0.617, *p* < 0.001). Xu et al. (2024) similarly identified organizational agility as a significant mediator between digital transformation and innovation performance, and Wamba (Fosso Wamba, 2022) reported a comparable mediating role for organizational agility between AI assimilation and firm performance. These studies rely on related rather than identical outcome constructs, yet they point to the same underlying mechanism: AI-enabled or digital capabilities feed into organizational outcomes partly through agility. The present study builds on this evidence by pinning down indirect-only mediation for the AAA–FA–FCA relationship under the BCa confidence-interval rule.

The predictive-relevance results round out these explanatory findings. Blindfolding yielded *Q^2^* = 0.224 for FA and *Q^2^* = 0.374 for FCA, both above zero and thus indicating predictive relevance for the two reflectively measured endogenous constructs. FCA’s larger *Q^2^* fits with the model’s strong FA → FCA relationship and reinforces the practical relevance of the downstream value-realization pathway—though *Q^2^* should be read as an in-sample predictive-relevance criterion rather than proof of out-of-sample prediction, and it remains conceptually separate from *R^2^*, effect sizes, and bootstrap significance tests (Hair et al., 2023; Hair et al., 2019).

H1’s coefficient is positive (*β* = 0.146) with a *p*-value under 0.05, yet its BCa 95% confidence interval [−0.002, 0.266] spans zero—so under the confidence-interval rule adopted here, H1 is not supported. A positive point estimate is still consistent with infrastructure playing a helpful role. Still, its independent effect loses stability once data, human, cultural, task, and analytical-capability fit all enter the picture together. One plausible reading is that infrastructure functions as a foundational or threshold resource: necessary for augmented analytics to operate at all, but not something that further differentiates adoption once a baseline level of technological readiness is met. This fits with RBV and analytics-capability research more broadly, which stresses resource combinations and deployment over the mere possession of technology (Arunachalam et al., 2018; Côrte-Real et al., 2017; Muslimin et al., 2017; Gupta and George, 2016; Grover et al., 2018; Seddon et al., 2017; Alghamdi and Al-Baity, 2022; Oesterreich et al., 2021).

H2 held up (*β* = 0.188; BCa 95% CI [0.058, 0.339]), indicating that the closer the data environment matches the requirements of augmented analytics, the more likely adoption becomes. Automated models inherit whatever accessibility, integration, relevance, and quality limits exist in their input data, so data fit is more than a technical convenience—it is a precondition for generating credible insight. This aligns with evidence that data quality and data-related capabilities underpin analytics use, innovation, agility, and business value (Ghasemaghaei and Calic, 2019; Ashrafi et al., 2019; Gupta and George, 2016; Wang et al., 2018; Grover et al., 2018; Ren et al., 2017; Alghamdi and Al-Baity, 2022), and helps explain why infrastructure alone falls short: computing power cannot make up for data that are fragmented, inaccessible, or simply unsuited to the analytical task at hand.

H3 was also supported (*β* = 0.150; BCa 95% CI [0.012, 0.309]), suggesting that adoption is more likely where employees’ analytical knowledge and skills match the demands of augmented analytics. This backs a human-augmentation reading of AI-enabled analytics: automation may widen access to models and insights, but people still have to frame the right questions, judge plausibility, interpret context, and turn outputs into decisions (Prat, 2019; Jarrahi, 2018; Ammenwerth et al., 2006; Ghasemaghaei et al., 2017; Alghamdi and Al-Baity, 2022; Salazar and Kunc, 2025). Human capability, then, complements automation rather than becoming a residual requirement that fades as automation increases.

H4 did not hold up in the revalidated analysis (*β* = 0.047, *p* = 0.467; BCa 95% CI [−0.100, 0.163])—a result that refines rather than undermines TTF. Classical TTF theory expects technology to align with task requirements (Goodhue and Thompson, 1995; Goodhue, 1998). Still, once data quality, user capability, culture, infrastructure, and analytics capability are all entered together, task fit can end up sharing explanatory ground with these more proximal conditions, or may be necessary without being sufficient on its own. A function can look well matched to a reporting task on paper and still go underused in practice if the underlying data are unreliable, users cannot make sense of the output, or organizational routines fail to support evidence-based action (Ghasemaghaei et al., 2017; Daradkeh, 2019; Alghamdi and Al-Baity, 2022; Oesterreich et al., 2021). Read this way, the unsupported path is informative in its own right: it shows that adoption cannot be reduced to task compatibility alone.

H5 was supported (*β* = 0.141; BCa 95% CI [0.014, 0.270]), indicating that adoption comes more easily where organizational norms and practices already align with data-driven, AI-supported decision-making. Because augmented analytics can surface unexpected patterns and challenge entrenched judgments, its use demands a culture that tolerates evidence-based pushback, learning, experimentation, and cross-functional collaboration—consistent with culture-technology fit and analytics-value research that treats organizational routines and acceptance as complements to technical capability (Lee et al., 2007; Grover et al., 2018; Seddon et al., 2017; Alghamdi and Al-Baity, 2022; Oesterreich et al., 2021).

H6 was not only supported but produced the largest coefficient among the six fit antecedents (*β* = 0.219; BCa 95% CI [0.077, 0.352]), pointing to a clear benefit from prior analytical capability that lets organizations fold augmented tools into existing data, modeling, visualization, and decision routines. This tracks with RBV: new technology tends to gain traction faster when it can be layered onto existing valuable knowledge and processes (Ghasemaghaei et al., 2017; Warth et al., 2011; Gupta and George, 2016; Grover et al., 2018; Seddon et al., 2017). It also sets augmented analytics apart from a plug-and-play application—AI-enabled functionality can automate parts of the lifecycle, but organizations still need the absorptive and analytical capacity to evaluate, govern, and put its outputs to use (Prat, 2019; Alghamdi and Al-Baity, 2022; Oesterreich et al., 2021; Salazar and Kunc, 2025).

A clear pattern runs through the downstream structural relationships. AAA relates strongly and positively to firm agility (H7a: *β* = 0.625; BCa 95% CI [0.533, 0.697]), and firm agility relates strongly and positively to competitive advantage (H7b: *β* = 0.712; BCa 95% CI [0.636, 0.791]); by contrast, the direct AAA → FCA path is small, with a BCa interval that includes zero (H7c: *β* = 0.097; BCa 95% CI [−0.005, 0.179]). So H7a and H7b hold up, while H7c does not survive the BCa interval rule. This asymmetry makes theoretical sense: rival firms can acquire comparable analytical technologies, but they differ in how well they reorganize decisions and actions around the insights those technologies produce. What proves hard to imitate, then, is not adoption itself but the coordinated sensing-and-response process that agility represents (Ashrafi et al., 2019; Ghasemaghaei et al., 2017; Peters et al., 2016; Atienza-Barba et al., 2024; Xu et al., 2024; Abdul Wahab and Radmehr, 2024; Mariani and Mancini, 2025; Sadeghi R et al., 2024; Hyun et al., 2023).

H8 is supported: the specific indirect AAA → FA → FCA effect comes to *β* = 0.445 (BCa 95% CI [0.373, 0.521]), against a total AAA → FCA effect of *β* = 0.542. This indirect-only pattern (Preacher and Hayes, 2008; Nitzl et al., 2016; Zhao et al., 2010) offers a specific account of how organizational value is realized—AAA sharpens the speed and breadth of analytical sensing. At the same time, firm agility lets the organization mobilize that information into faster decisions and adaptive action. A mechanism-based result of this kind carries more theoretical weight than a bare direct-effect claim, since it pinpoints the organizational capability through which augmented analytics connects to competitive advantage (Seddon et al., 2017; Atienza-Barba et al., 2024; Xu et al., 2024; Abdul Wahab and Radmehr, 2024; Mariani and Mancini, 2025; Sadeghi R et al., 2024; Salazar and Kunc, 2025; Hyun et al., 2023).

Across the 287-case analysis, H2, H3, H5, H6, H7a, H7b, and H8 were all supported. H1 did not clear the BCa confidence-interval rule despite a positive coefficient and *p* < 0.05; H4 was not supported; and the direct H7c path did not hold either. Far from being a shortcoming, these unsupported paths carry information of their own—they rule out the assumption that all resources matter equally. What emerges instead is a leaner structure in which credible data, capable users, a supportive culture, and established analytical capability are the clearest conditions for adoption, with firm agility standing out as the main mechanism by which value gets realized. Reporting the unsupported paths alongside the supported ones also keeps the analysis transparent. It preserves the model’s ability to separate robust effects from relationships that are theoretically plausible but empirically shaky.

### 5.2 Theoretical implications

The first theoretical implication concerns fit itself. Rather than a single task-function correspondence, the findings extend TTF into a portfolio of resource-technology alignments. The uneven coefficients show that fit is not a one-size-fits-all organizational condition: data, analytics capability, individual capability, and culture relate more clearly to AAA than task fit does, while infrastructure looks more like an enabling resource than a differentiating one. This multidimensional pattern sharpens conventional TTF reasoning by showing that AI-enabled analytics adoption depends on jointly aligning technical, informational, human, and cultural resources (Prat, 2019; Ghasemaghaei et al., 2017; Daradkeh, 2019; Lee et al., 2007; Warth et al., 2011; Goodhue and Thompson, 1995; Goodhue, 1998; Alghamdi and Al-Baity, 2022; Oesterreich et al., 2021).

A second implication bears on RBV. The results favor a configurational reading in which resources create value through complementarity and actual use, rather than mere possession. Data fit and analytics-capability fit relate more clearly to adoption than infrastructure fit does, which suggests that widely available technology is not, on its own, a sustained strategic resource. Suitable data, established analytical routines, skilled users, and a supportive culture, taken together, are harder to assemble and imitate—and so fit more comfortably with an RBV account of why organizational outcomes differ across firms (Arunachalam et al., 2018; Côrte-Real et al., 2017; Muslimin et al., 2017; Gupta and George, 2016; Grover et al., 2018; Seddon et al., 2017; Wamba et al., 2015).

The model’s third and central theoretical contribution concerns dynamic capabilities. Firm agility provides the strongest link between AAA and competitive advantage: the supported indirect effect, paired with the unsupported direct effect, shows that analytical adoption translates into advantage mainly when an organization can convert insight into rapid sensing, decision-making, and reconfiguration. In this way, the study connects two stages usually studied apart—technology adoption and organizational value realization—with agility positioned as the bridge between them (Ashrafi et al., 2019; Ghasemaghaei et al., 2017; Atienza-Barba et al., 2024; Xu et al., 2024; Abdul Wahab and Radmehr, 2024; Mariani and Mancini, 2025; Sadeghi R et al., 2024; Hyun et al., 2023). This mechanism also explains why technically similar investments can leave firms with quite different competitive outcomes.

### 5.3 Practical implications

For managers, the selective pattern in the antecedent results points toward a staged readiness assessment rather than a technology-first rollout. With data-technology fit and analytics-technology fit both supported, organizations should start by documenting critical data sources, assigning clear data ownership, setting quality rules, clearing access barriers, and consolidating fragmented repositories before scaling up augmented-analytics use cases. Dashboards that track completeness, accuracy, timeliness, lineage, and exceptions can keep data readiness visible and governable throughout.

Because individual-technology fit was supported, human capability should rank as a second implementation priority. Training programs should cover analytics literacy, question formulation, interpreting AI-generated insights, handling uncertainty, and knowing when to escalate implausible outputs. Firms can pair role-based instruction with supervised use cases, where analysts, domain specialists, and managers jointly assess whether an insight is credible and operationally relevant—treating human judgment as a complement to automation rather than assuming self-service tools eliminate the need for analytical skill.

The supported culture-technology fit path calls for changes to decision routines alongside training. Senior managers should ask that evidence accompany major proposals, allow room for structured experimentation, and set up cross-functional forums where technical and business teams can question model outputs without discouraging adoption. That task-technology fit came back unsupported does not mean tasks are beside the point—it signals that even a well-matched use case can still fail if the surrounding data, skills, analytical routines, or decision norms are weak. Selecting use cases should therefore weigh the task together with the organizational conditions that surround it.

Analytics capability is best built by folding augmented functions into existing BI and analytical workflows rather than standing up an isolated platform. Organizations can set review and approval points for automated insights, clarify who is accountable for models and data, and connect analytical outputs directly to operational systems and decision rights. Given the strong AAA → FA and FA → FCA paths, implementation should also aim to shorten the gap between insight and action—through mechanisms such as delegated decision thresholds, rapid-response teams, preapproved response playbooks, and feedback loops that track whether an insight actually changed a decision or process.

Progress is best tracked through a balanced set of adoption and agility indicators—time-to-insight, decision-cycle time, frequency of analytical use, active-user and use-case adoption rates, the share of generated insights reviewed, the share converted into documented actions, response time following a material insight, and data-quality exception rates. Metrics like these separate software installation from genuine effective use, linking the supported fit dimensions and the agility pathway to outcomes managers can observe.

### 5.4 Strengths and shortcomings of the model

One strength of the model lies in combining several resource-technology fit dimensions with a downstream organizational capability, which makes it possible to separate the conditions associated with adoption from the mechanism through which adoption translates into competitive advantage. Modeling AAA formatively reinforces this logic, treating adoption as something constituted by complementary dimensions rather than expressed through interchangeable indicators of a single trait.

The model has its limits, too. The six fit antecedents cannot capture every organizational condition that might shape AI-enabled analytics, and the cross-sectional design leaves temporal causality unestablished. The formative AAA specification could not be paired with a redundancy analysis, since the archived dataset lacked a separate global criterion item, and several HTMT values sit close to the conventional 0.90 boundary. For this reason, blindfolding *Q^2^* was limited to the reflectively measured endogenous constructs FA and FCA; while the positive *Q^2^* values speak to predictive relevance, they should not be mistaken for a dedicated out-of-sample prediction test. None of this invalidates the model, but each point marks an area where future replication could strengthen measurement and prediction.

### 5.5 Limitations and future research

The study carries several limitations. The cross-sectional data mean the temporal order of these relationships cannot be pinned down, so while the mediation results are consistent with the proposed model, they should be read as evidence of an indirect association rather than proof of longitudinal causality.

Purposive non-probability sampling was used to reach respondents with genuine analytics experience—useful for obtaining well-informed responses, but it meant the probability of selection was never known. Accordingly, the sample should not be treated as statistically representative of Jordanian organizations as a whole.

The study was carried out in Jordan specifically, and the institutional, cultural, economic, technological, and regulatory context there may shape the strength of the modeled relationships. Replicating the study across other countries and sectors—ideally with probability-based samples where feasible—would strengthen the evidence for external validity and clarify the model’s boundary conditions.

All the main constructs were measured through the same self-report questionnaire. The full-collinearity VIF diagnostic turned up no sign of severe CMB, but the common-source design still counts as a limitation; future studies might draw on multiple respondents, objective indicators, temporal separation, or marker-variable techniques instead.

The model itself is confined to six fit dimensions plus firm agility. Other factors—top-management support, AI governance, data security, privacy, leadership, financial resources, and environmental turbulence, among them—could well shape adoption or the value organizations ultimately draw from it.

The archived SmartPLS bootstrapping run used 500 subsamples, and the results here are reported exactly as that traceable run produced them, rather than adjusted after the fact. Given that the H1 and H7c BCa confidence limits sit close to zero, a future replication using a larger bootstrap run would help clarify how stable these borderline intervals really are.

Longitudinal designs would also be a natural next step, testing whether shifts in resource-technology fit actually precede shifts in augmented analytics adoption, and whether changes in turn follow adoption in agility and competitive outcomes.

## 6 Conclusion and potential future work

Taken as a whole, this study shows that the organizational value of augmented analytics builds up through a sequence of events rather than arriving with a single adoption decision. Among the six resource-technology fit dimensions, data fit, individual capability fit, culture fit, and analytics-capability fit relate most clearly to adoption. In contrast, task-technology fit and the IT-infrastructure path fall short under the BCa confidence-interval rule. Technology readiness alone is not enough; adoption tracks more closely with the quality of the informational, human, analytical, and cultural resources surrounding it.

The central conclusion concerns what happens downstream. AAA relates strongly to firm agility, and firm agility relates strongly to competitive advantage, while the direct AAA → FCA path does not hold up. The supported indirect effect thus marks agility as the principal mechanism connecting augmented analytics adoption to competitive advantage. Blindfolding results (*Q^2^* = 0.224 for FA and 0.374 for FCA) add further evidence of predictive relevance for these outcomes, though they do not substitute for genuine out-of-sample prediction.

The study’s contribution lies in bringing three explanatory levels together in one model: multidimensional fit accounts for the conditions tied to adoption, RBV explains why complementary resources matter more than simply owning technology, and dynamic capability theory explains how adoption converts into organizational value through agility. The unsupported paths sharpen this contribution further, showing that neither fit alone nor technology adoption alone produces equivalent outcomes across organizations.

Future work should test the temporal ordering of this model through longitudinal designs, replicate it across countries and sectors, and bring in governance, leadership, privacy, data security, and environmental turbulence. It would also help to use larger bootstrap resampling runs, add a global criterion item for formative redundancy assessment, and pair blindfolding with a dedicated out-of-sample prediction procedure—extensions that would test how robust the value-realization mechanism identified here really is, and where its boundaries lie.

## Data Availability

The original contributions presented in the study are included in the article/Supplementary material, further inquiries can be directed to the corresponding authors.
